# Influence of Fever on a Case of Traumatic Tetanus

**Published:** 1896-01

**Authors:** A. S. Wheeler

**Affiliations:** New Orleans, La.


### THE INFLUENCE OF FEVER ON A CASE OF TRAUMATIC TETANUS.

By A. S. WHEELER, V.M.D.,
NEW ORLEANS, LA.

On April 10, 1895, I was called to see a five-year-old mule that was suffering from tetanus, caused by a nail-wound of three days' duration. From the day of my first visit the case grew steadily worse, until the seventh (April 17th) day, whereupon I told the owner that there was but the slimmest chance for the animal; the jaws were almost immovable, and the poor beast was scarcely able to stand from the tension of the abductors. I had him placed on slings and made as comfortable as possible, leaving instructions to notify me by telephone the next morning if the mule died during the night. Having received no message the next morning, I went to see the patient again, and to my astonishment found him better as to the tetanus, but a slight cough and fever (102°) had sprung up. On the following day the temperature reached 104°–105°; the jaws were still more relaxed, and there was at this time considerable discharge from both nostrils, as if a distemper was supervening. In spite of the reduction of the febrile symptoms with antipyretics and inhalations of carbolized vapor, gangrene of the lungs developed, and the animal succumbed on April 26th from septicæmia. Every vestige of tetanus had disappeared several days before death.

The soothing influence of fever on nervous diseases has often been recognized, especially in insanity, and the laxative effect of pyrexia in the case I have just cited was almost magical.

---

Prof. James Law, of Cornell University, was a visitor to to Philadelphia, inspecting the Veterinary Department of the University of Pennsylvania and the Philadelphia Veterinary Sanatarium, in order to combine in the new school buildings of the New York State Veterinary College all the best features known in the construction of such institutions.

Prof. R. S. Huidekoper, of New York City, is entertaining a proposition to assume the directorship of a very large breeding and stock-raising enterprise in Virginia. Besides aiming to produce fine stock of several species and breeds, attention being given also to poultry, etc., the undertaking has in view the production of high grades of flesh and fowl for the table and food-markets. The enormous consumption of these products, associated with the movement, will allow for a constant weeding out of inferior specimens for breeding purposes and thus enable the projectors to rapidly attain the highest state of perfection, with profit on both ends.

